# A comparison of reward processing during Becker–DeGroot–Marschak and Vickrey auctions: An ERP study

**DOI:** 10.1111/psyp.14313

**Published:** 2023-04-19

**Authors:** A. Newton‐Fenner, D. Hewitt, J. Henderson, N. Fallon, Y. Gu, O. Gorelkina, T. Giesbrecht, A. Stancak

**Affiliations:** ^1^ Department of Psychology University of Liverpool Liverpool UK; ^2^ Institute of Risk and Uncertainty University of Liverpool Liverpool UK; ^3^ Wellcome Centre for Integrative Neuroimaging University of Oxford Oxford UK; ^4^ Management School University of Liverpool Liverpool UK; ^5^ Henley Business School University of Reading Reading UK; ^6^ Unilever, Research and Development Port Sunlight UK

**Keywords:** EEG, N170, P3, reward, RewP, subjective value, willingness‐to‐pay

## Abstract

Vickrey auctions (VA) and Becker–DeGroot–Marschak auctions (BDM) are strategically equivalent demand‐revealing mechanisms, differentiated only by a human opponent in the VA, and a random‐number‐generator opponent in the BDM. Game parameters are such that players are incentivized to reveal their private subjective values (SV) and behavior should be identical in both tasks. However, this has been repeatedly shown not to be the case. In this study, the neural correlates of outcome feedback processing during VA and BDM were directly compared using electroencephalography. Twenty‐eight healthy participants bid for household products which were then divided into high‐ and low‐SV categories. The VA included a human opponent deception to induce a social environment, while in reality a random‐number‐generator was used in both tasks. A P3 component peaking at 336 ms over midline parietal sites showed more positive amplitudes for high bid values, and for win outcomes in the VA but not the BDM. Both auctions also elicited a Reward Positivity potential, maximal at 275 ms along the central midline electrodes, that was not modulated by auction task or SV. Further, an exploratory N170 potential in the right occipitotemporal electrodes and a vertex positive potential component were stronger in the VA relative to the BDM. Results point to an enhanced cortical response to bid outcomes during VA task in a potential component associated with emotional control, and to the occurrence of face‐sensitive potentials in VA but not in BDM auction. These findings suggest modulation of bid outcome processing by the social‐competitive aspect of auction tasks. Directly comparing two prominent auction paradigms affords the opportunity to isolate the impact of social environment on competitive, risky decision‐making. Findings suggest that feedback processing as early as 176 ms is facilitated by the presence of a human competitor, and later processing is modulated by social context and subjective value.

## Abstract 2

Directly comparing two prominent auction paradigms affords the opportunity to isolate the impact of social environment on competitive, risky decision‐making. Findings suggest that feedback processing as early as 176 ms is facilitated by the presence of a human competitor, and later processing is modulated by social context and subjective value.

## 1 INTRODUCTION

Social comparison and competition have been shown to affect reward‐seeking behavior, subjective valuation, and outcome processing during economic decision‐making (Bhanji & Delgado, 2014). Emphasis on competition during an auction can increase bid frequency (Heyman et al., 2004; Kamins et al., 2011), overbidding (Delgado, Schotter, et al., 2008; Teubner, 2013; van den Bos et al., 2008) and the prevalence of “the winner's curse,” where the winning bid exceeds the worth of the auction item (Malhotra, 2010; Park & Bradlow, 2005). However, the neural mechanisms underlying these processes are underexplored.

Vickrey auctions (VA) and Becker–DeGroot–Marschak auctions (BDM) are two of the most widely used demand‐revealing mechanisms in experimental economics (Lucking‐Reiley, 2000; Noussair et al., 2004), and notably have been adopted by online auction websites such as eBay. In both auctions, the player who submits the highest bid for a given good wins the auction, but pays a price equal to the second‐highest bid (Vickrey, 1961). The only difference between the two is that in the VA, players compete against other human players, whereas in the BDM a single player bids against a random number generator (Becker et al., 1964). Unlike other auction structures, both the VA and BDM purport dominant strategies of bidding one's true subjective value (SV), as deviating from doing so risks paying more than they believe the item is worth or missing out on the item for a price they were willing to pay. Importantly, these strategies are impervious to the risk attitude of the player and the strategies of other players.

The vast majority of risky decision‐making tasks employ monetary gambling paradigms where the participant can win and lose varying amounts of currency. The VA and BDM are different in that, in economic terms, the outcomes are either good (in the case of win) or neutral (in the case of no‐win); the degree to which a given win outcome is good depends on the difference between the bid value and the final price paid. In the BDM, outcomes are processed as purely economic rewards and can be ranked on that single dimension. Meanwhile, the VA involves the additional dimension of social value, which is a combination of the validation of shared public values and the value of “winning” as a separate entity to the value of the item won (Ariely & Simonson, 2003; Astor et al., 2013; Delgado, Schotter, et al., 2008; Malhotra, 2010).

Further, the BDM is formally strategically equivalent to a VA against a single unknown bidder, who bids their valuation, and whose value is drawn from the same distribution of valuations as that of the BDM prices. Therefore theoretically, under these conditions, the BDM and VA paradigms should elicit the same responses in players. However, many behavioral studies have found significant heterogeneity in bidding behavior in the VA compared to the BDM (Irwin et al., 1998; Kagel et al., 1989; Kagel & Levin, 1993; Noussair et al., 2004). Despite clear instructions and a full understanding of the paradigm, underbidding, and overbidding relative to SV are common in the VA (Flynn et al., 2016; Georganas et al., 2017; Noussair et al., 2004; Rosato & Tymula, 2019). The deviation from logical decision‐making has been attributed to several factors, including: feelings of spite induced by competition (Bartling et al., 2017; Kagel et al., 1989; Kagel & Levin, 1993; Ku et al., 2005), the “joy of winning” and the fear of losing (Astor et al., 2013), and differences in risk and uncertainty between the two paradigms (Levy et al., 2010; Noussair et al., 2004). The direct comparison of the VA and BDM under these conditions affords the opportunity to isolate the impact of a social environment and competition on decision‐making processes in the brain.

The inclusion of a second player in the VA has several implications. Whereas the BDM places players in a situation of individual choice, in the VA the player is now being observed by a competitor and can utilize their opponent's bid values to inform them about the items' common/public value (Toelch et al., 2014). The items also now have an additional dimension of value, in that the act of winning against another person holds a worth that is separate from the value of the item itself (Noussair et al., 2004). Further, while both tasks place players in a situation of decision‐making under uncertainty, in the BDM players are in a situation of static risk, where the computer bid values have equal probability across the entire range. Therefore, participants can quantify the probability of winning any given trial depending on their bid value. However, in the VA, the human opponent is unpredictable, in that opponent bids will not be equally distributed across the entire range of values. As a consequence, the players are placed in a situation of uncertainty, where they cannot gauge the likelihood of a given outcome based on their bid (Levy et al., 2010). Therefore, we would expect there to be a difference in subjective outcome probability expectation between BDM and VA.

Several studies investigating SV and outcome processing during decision‐making have found increased arousal and immediate emotional responses in the presence of a human opponent, as evidenced by increased heart rate and skin conductance responses (Adam et al., 2015; Astor et al., 2013; Teubner et al., 2015), and stronger activations in brain regions related to emotion processing (Delgado, Li, et al., 2008; Sanfey et al., 2003), social preferences (Sanfey et al., 2003; van den Bos et al., 2013), and mentalizing (Riedl et al., 2011).

Reward positivity (RewP), also known as feedback‐related negativity (FRN), is the most widely investigated event‐related potential (ERP) component in the outcome processing stage (Falkenstein et al., 1991; Walsh & Anderson, 2012). The RewP is maximal at 200–300 ms post‐feedback onset over frontocentral sites and reflects a subjective reward prediction error signal. It is characterized by a suppressed negative deflection elicited by win outcomes, that is not present in loss outcomes, giving the appearance of enhanced negativity for bad outcome feedback (Hakim & Levy, 2019; Holroyd et al., 2004; Miltner et al., 1997; Nieuwenhuis et al., 2004). The RewP is also dependent on the relationship between expected rewards and actual rewards, with increased amplitudes for unexpected compared to expected outcomes (Ferdinand et al., 2012; Hajcak et al., 2005; Hajcak et al., 2007; Pfabigan et al., 2011). The RewP is also sensitive to outcome magnitude (Goyer et al., 2008; Gu et al., 2011; Sambrook & Goslin, 2015) and salience (Hauser et al., 2014; Talmi et al., 2013; Walentowska et al., 2019; Yeung et al., 2005). However, the RewP was not sensitive to reward magnitude in our previous VA study (Newton‐Fenner et al., 2022), as characterized by auction item market value.

Notably, while a social dimension adds saliency to the context and induces comparison of one's task performance to others (Fehr & Schmidt, 1999; Fliessbach et al., 2007; Kedia et al., 2014), the evidence of an impact on RewP amplitudes is mixed. RewP amplitudes have been shown to be larger for non‐conformity when comparing performance to other players during a lottery task (Luo et al., 2015), and for competition compared to cooperation during a perceptual four‐alternative forced‐choice task (Czeszumski et al., 2019). However, no difference in RewP amplitudes was found comparing non‐social, socially comparative, and socially competitive conditions during a monetary gambling task (Rigoni et al., 2010). We hope to shed light on this inconsistency, using a context where the participant's optimal strategy to maximize their payoff is the same, regardless of the social context. As the level and type of competition remain the same between tasks, we can delineate the effect of competition from the social environment.

The P3 (or P300) component, a large positive deflection elicited 300–450 ms along midline central‐parietal electrodes post‐feedback onset (Polich, 2007, 2012) is also extensively studied during outcome processing and performance monitoring. The P3 is thought to be central to indexing attention for novel stimuli (Schuermann et al., 2012), mismatch detection, and context updating (Martin & Potts, 2011), and is highly sensitive to the motivational significance of stimuli (Nieuwenhuis, Aston‐Jones, & Cohen, 2005; San Martin, 2012; Yeung & Sanfey, 2004). Regarding reward processing, positive feedback elicits larger P3 amplitudes compared to negative feedback (Pfabigan et al., 2011), as do reward outcomes associated with higher levels of arousal or task relevance, reflecting an increased allocation of attention (Nieuwenhuis et al., 2011). The P3 is also sensitive to outcome magnitude, with larger rewards/losses eliciting greater amplitudes (Bellebaum & Daum, 2008; Yeung & Sanfey, 2004). Relevantly, in our most recent study using a VA task, auction outcomes were associated with larger P3 amplitudes for high‐market value than low‐market value items, and win outcomes compared to no‐win outcomes (Newton‐Fenner et al., 2022). Similarly, Tyson‐Carr et al. (2018) also found during a BDM task that high‐value products produced increased activation in the P3 interval during the initial valuation period.

This study focused on the time course of neural activity during feedback processing upon receipt of the auction outcome. We analyzed the amplitudes of the RewP and P3 components during the outcome processing period to gain insight into the temporal progression of attentional biases and attentional resource assignment, preference encoding, prediction error, and motivation during decision‐making. We hypothesized that RewP amplitudes would differentiate between the BDM and VA tasks, but not between high‐ and low‐value items as denoted by bid value. The P3 component was predicted to be larger for wins than no‐wins, and for high‐value than low‐value items, in both tasks, in line with our previous findings. In addition to the hypothesized components, during visual inspection of the topographies, we also found an enhanced negative potential in the right occipitotemporal electrodes and a vertex positive potential (VPP) in the latency of 170–180 ms during the VA compared to the BDM. The configuration is consistent with the face‐sensitive N170 ERP component occurring during viewing human faces (Deffke et al., 2007; Dijkstra et al., 2018; Rossion, 2014; Rossion & Jacques, 2012; Weiß et al., 2020), and so we performed a post hoc exploratory analysis.

This is the first electroencephalography (EEG) study to directly compare the neural correlates of decision‐making during the VA and the BDM. Contrasting the two paradigms isolates the effect of a competitive environment on ERPs while keeping the paradigm identical in all other respects. This informs work on SV, reward processing, overbidding, and competitive arousal.

## 2 METHOD

### 2.1 Participants

Twenty‐four healthy, right‐handed participants (14 female) with a mean age of 25.9 ± 5.4 years (±SD) completed the study. Four additional participants (3 female) were removed prior to ERP analysis due to not properly following the study procedure. All participants had normal or corrected‐to‐normal vision and were screened for psychological/psychiatric disorders. All gave written informed consent and were reimbursed for their time and travel expenses. The experimental procedures were approved by the Research Ethics Committee of the University of Liverpool and were in accordance with the Declaration of Helsinki.

### 2.2 Procedure

The study was carried out over two sessions separated by a minimum of 7 days (mean 10.3). Participants completed computerized VA and BDM tasks, one task per session, while brain activity was recorded with EEG. The order of the tasks was counterbalanced. The purpose of the experiment and instructions for the tasks were explained to participants at the beginning of the session. All experimental procedures were carried out in a dimly lit, sound‐attenuated Faraday cage. Both tasks were displayed on a 19‐inch LED monitor using PsychoPy software (Peirce et al., 2019).

#### 2.2.1 BDM task

**FIGURE 1:**
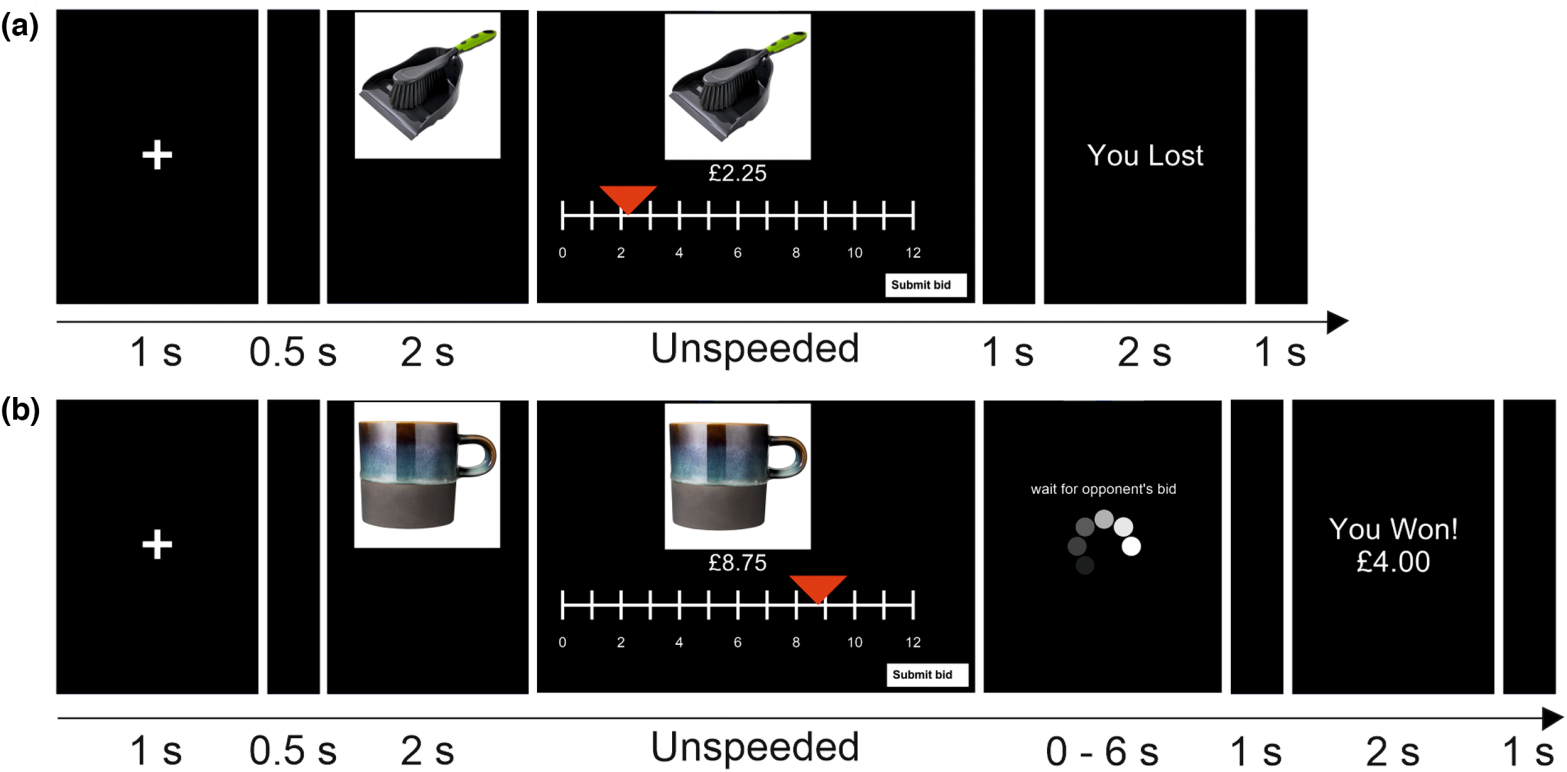
(a) A no‐win trial in the BDM task. b) A win trial for the VA task. For both tasks, each trial began with a fixation cross for 1 s, followed by the auction item for 2 s, which is then joined by a sliding scale from £0 to £12 in increments of 25p on which to select their bid. Participants were instructed to select their bid on the scale, and once they were happy with their decision, to submit the bid by clicking on the button in the bottom right‐hand corner. In the VA, in two‐thirds of the trials, this was followed by the phrase “wait for opponent” and a loading GIF indicating that the other player has yet to submit their bid, which lasted for either 1–2 or 5–6 s. This was then followed by a blank screen for 1 s. In the BDM, the screen was blank for 1 s before presenting the outcome of the trial. The outcome of the trial was then presented for 2 s. EEG triggers were synced to the onset of auction feedback.

The BDM task protocol can be seen in Figure 1. At the beginning of the session, participants were informed that they would be bidding against a random number generator in a computerized auction task. If the participant bid higher than the random number, they would win the item and pay the random number as the price; if they bid lower than the random number they would lose the trial, not winning the item and paying nothing. In the case of both bids being equal, the winner was decided randomly.

Participants received an initial endowment of £12 and were instructed to use it to purchase items during the BDM task. They were informed that at the end of the session, one of their winning trials would be randomly selected and the price that they won the item for would be deducted from their endowment; they would receive the remaining amount of their endowment and the item as reimbursement for their participation. After the application of the EEG net, participants were led into the Faraday cage to complete the task. Participants were seated in front of the computer and rested their dominant hand on a computer mouse.

The protocol for the BDM task was adapted from previous studies (Kokmotou et al., 2017; Roberts et al., 2018; Tyson‐Carr et al., 2018; Tyson‐Carr et al., 2020). The stimuli comprised 300 everyday household products such as kettles, batteries, and mugs, valued in the ranges £3–7 (low‐value) and £8–12 (high‐value; *n* = 150 in each range), with a mean value of £7.39 ± £3.12 (±SD) obtained from a shopping catalog. Efforts were made to distribute retail prices evenly within the two ranges. Each auction trial began with a resting interval where participants viewed a white fixation cross on a black background for 2 s. The participants were then presented with an item to bid on, using a sliding scale from £0 to £12 in increments of 25p. The item was presented for 2 s before the sliding scale appeared. Participants selected their bid using the sliding scale and submitted the bid by clicking on a button in the bottom right‐hand corner. There was no time limit on bid submission and participants could click on the scale as many times as they wished before submitting their bid. The opponent bid was generated by the random function in Excel (Microsoft, USA) to be drawn from a uniform distribution between £0 and £12 with matched increments of 25p and compared to the submitted bids. The outcome was displayed on the screen (e.g. “you won! £2.75” or “you lost”) for 2 s.

Participants were instructed and confirmed that they understood, that there was an equal likelihood of the random number bidding any increment of 25p between £0 and £12, and so their bid directly corresponded to the likelihood that they would win a given trial. For example, if they place a bid of £6 on an item, the likelihood of them winning is 50%. The order of item presentation was randomized, and each item was presented once, resulting in a total of 300 auction trials. The task was broken up into five blocks of 60 trials, and participants were given a short break in between blocks to limit fatigue and to make any necessary adjustments to the EEG system. The duration of each block was approximately 12 min.

#### 2.2.2 VA task

The VA task protocol can be seen in Figure 1. The VA task was identical to the BDM task in all but two respects. First, at the beginning of the session, the participants were informed that they were playing against another human participant as their opponent, who was situated in another room so they would remain anonymous. They were told that for each trial, whoever bid higher would win the item but pay the price equal to their opponent's bid. In reality, the participants were bidding against the random number generator. Second, a jitter of between 0 and 6 s was included post‐bid submission where the phrase “wait for opponent” and a loading GIF appeared on screen to mimic a human opponent deciding on their bid. Participants were informed of the deception during the debriefing. All participants confirmed that they believed that they were bidding against another person during the VA during an informal exit interview. The same stimuli, trial number, and timings were used in the VA task as in the BDM.

### 2.3 EEG recordings

EEG was recorded continuously using a 129‐channel Geodesics EGI System (Electrical Geodesics, Inc.) with a sponge‐based HydroCel Sensor Net. The sensor net was aligned with respect to three anatomical landmarks: two preauricular points and the nasion. Electrode‐to‐skin impedances were kept below 50 kΩ and at equal levels across all electrodes, as recommended for the system (Ferree et al., 2001; Luu et al., 2001; Luu et al., 2003; Picton et al., 2000). The recording band‐pass filter was 0.001–200 Hz with a sampling rate of 1000 Hz. The electrode 129 (corresponding to Cz in the 10–10 system) served as the reference.

#### 2.3.1 ERP analysis

EEG data were pre‐processed with the BESA v. 7.0 program (MEGIS). EEG signals were spatially transformed to reference‐free data using the common average reference method (Lehmann et al., 1987). This spatial transformation restored the signal at electrode 129 for use in further analyses.

Ocular and electrocardiographic artifacts were removed using a combination of a pattern‐search algorithm and principal component analysis based on averaged eye blinks and artifact topographies (Berg & Scherg, 1994; Ille et al., 2002). Data were also visually inspected for the presence of atypical electrode artifacts, head movement artifacts, and artifacts related to muscle contractions. Continuous data were sectioned into epochs of 1200 ms duration each with a baseline interval ranging from −200 ms to 0 ms relative to feedback onset. Epochs contaminated with artifacts were manually excluded.

The average number of accepted trials after artifact exclusion was 254.2 ± 39.6 (mean ± SD) in the BDM and 260.5 ± 25.6 in the VA. For each condition, the average accepted number of trials were: in the BDM, high 127.7 ± 22.7; low 126.5 ± 18.3, win 101.2 ± 31.2; no‐win 153 ± 35.7; and in the VA, high 130.7 ± 15.5; low 129.8 ± 13, win 105 ± 23.6; no‐win 155.4 ± 31.6.

Conditions in the auction outcome period were grouped by value or outcome for statistical analysis. Paired t‐tests revealed that the average number of accepted trials differed between the win and no‐win conditions in both the BDM (win: 101.2 ± 31.2, no‐win: 153.0 ± 35.7) and VA (win: 105.0 ± 23.6, no‐win: 155.4 ± 31.6) (*p* < .001), but did not differ between high‐ and low‐value conditions for both time periods and both tasks. There were also no differences between the average number of accepted trials when conditions were compared across the two tasks (*p* > .05).

Data were filtered from 0.5 to 30 Hz and exported to EEGLab (Delorme & Makeig, 2004) for further processing. ERPs in response to outcome feedback were computed separately for each condition by averaging respective epochs in intervals ranging from 200 ms before outcome feedback onset to 1000 ms post‐feedback onset. EEG epochs were averaged for both tasks (VA and BDM), each type of outcome (win and no‐win), and both subjective value categories (high and low). The RewP potential was quantified as a win–minus–no–win difference waveform in the outcome receipt period. Based on visual inspection of scalp topographies and previous research (Glazer et al., 2018; Hauser et al., 2014; Krigolson, 2018; Meadows et al., 2016; Walsh & Anderson, 2012), the electrodes 129, 55 and 6 for the P3 and RewP components (corresponding to Cz, CPz, and FCz in the International 10–10 system, respectively), and 101, 100, 99 for the N170 component (electrode 100 corresponding to TP10 in the International 10–10 system) were selected for statistical analysis (Luu & Ferree, 2005). Time intervals of interest were selected based on visual inspection of waveforms in our data and according to the definitions concerning the time windows of each component from previous literature (Glazer et al., 2018; Polich, 2012; Roberts et al., 2019; Tyson‐Carr et al., 2018; West et al., 2014). These time periods were further analyzed using repeated measures ANOVAs. To compensate for violations of the sphericity assumption, a Greenhouse Geisser ɛ correction was used where applicable.

## 3 RESULTS

### 3.1 Behavioral results

The overall mean bid value across the two tasks was £4.61, with VA = £4.66 (SD ± 3.25) and BDM = £4.56 (SD ± 3.25). The participants were not told the retail price of the auction items so as not to anchor their bids. A Shapiro–Wilk test confirmed the bid values within the two tasks were normally distributed (*p* > .05). Response times were uninformative as judgments were not time limited. All but three participants showed a strong positive correlation between their bid values in the two tasks (*r* = 0.90–0.61); two showed a medium positive correlation (*s* = 0.431 and 0.367) and one a weak positive correlation (*r* = 0.273, *p* < .001). To explore the effects of high and low SV in subsequent ERP data analysis, the bid values for each participant were divided into low‐ and high‐value by the median split, with approximately *n* = 150 each and means of £2.15 (SE = 0.021) and £7.04 (SE = 0.039), respectively.

The effect of task order was investigated with paired samples t‐tests. Bid values in the first session were significantly higher than in the second session: *t*(299) = 4.896, *p* < .001, with a mean difference of 26p (SD ± 0.92). The order of the two tasks were counterbalanced; 10 participants bid more in the first session (6 in the VA and 4 in the BDM) and 10 participants bid more in the second session (5 in the VA and 5 in the BDM).

In order to determine whether participants changed their strategy as the tasks progressed, we correlated the average bid values in each task with the trial number. A small negative trend was observed in VA (*r(*22) = −0.118, *p* = .041), suggesting participants tended to decrease their bid as the task progressed. No significant correlation was found in the BDM.

Finally, overbidding or underbidding behaviors in the VA relative to the BDM were explored, however, there was no significant difference in bidding behavior between tasks (paired *t*‐test: *t* = −0.37, df = 23, *p* > .05). To examine any individual differences in bid behavior, a series of paired t‐tests compared the bid values for matched auction items during both tasks for each participant. Eleven participants bid more in the VA, 9 participants bid more in the BDM, and 4 participants' bid differences between tasks were not statistically significant. The overall average bid value across both tasks did not statistically significantly differ between VA > BDM and BDM > VA participants (*p* = .50). As there was no effect of strategy on the overall bidding behavior, strategy was not explored in the ERP analysis.

### 3.2 ERP results

**FIGURE 2:**
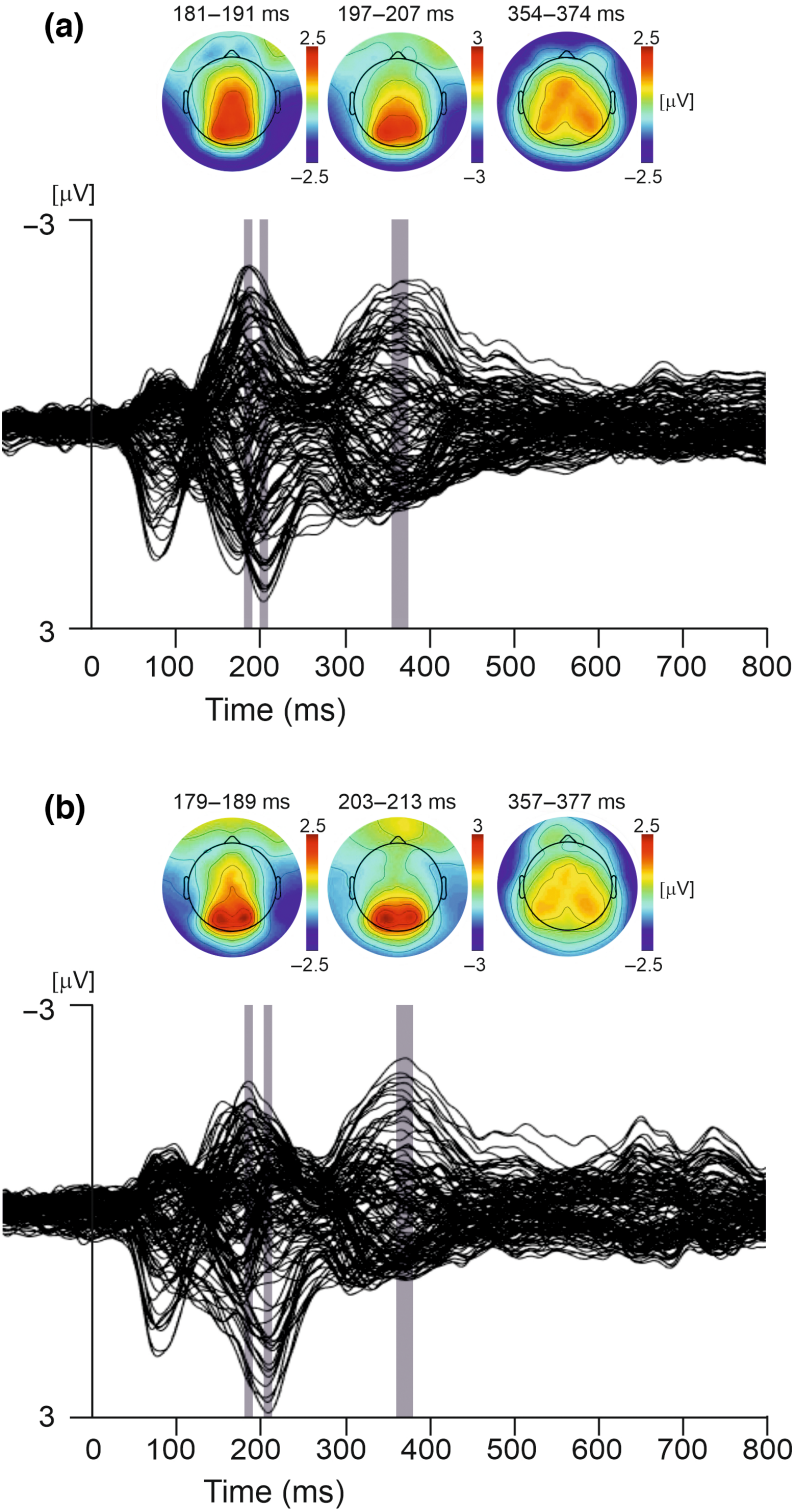
Butterfly plots of grand average ERPs in response to outcome presentation for (a) VA task and (b) BDM task. Epochs for distinct ERP components, N170, P2, and P3, are highlighted with gray bars, and the corresponding averaged topographies across the selected epochs are shown above.

Figure 2 shows a butterfly plot illustrating the ERPs at each electrode site in response to outcome presentation across all conditions and both tasks; ERP components and their corresponding latencies and topographic maps are labeled.

Three distinct ERP components were observed across the epoch. The RewP component was defined as the win–minus–no–win difference waveform and was measured from midline frontal‐central electrodes peaking at 275 ms post‐feedback onset. The P3 component (Polich, 2012) emerged at approximately 310 ms in a parietal region on the right side of the scalp, before reaching a positive maximum at 331 ms over the midline frontal electrodes.

The N170 component peaked at 182 ms and displayed a bilateral negative potential at occipitotemporal electrodes that was stronger on the right than the left side of the head. The prominent negative potential in the right occipitotemporal electrodes was accompanied by a vertex positive potential (VPP) (Figure 2). In contrast to the VPP seen in the VA task, the scalp potentials in the BDM task showed two symmetric positive spatial maxima in occipital regions of the scalp. The topographic maps of the VPP and N170 components overlaid on the 3D volume rendering of a human head are shown in Figure S1.

#### 3.2.1 N170 component

**FIGURE 3:**
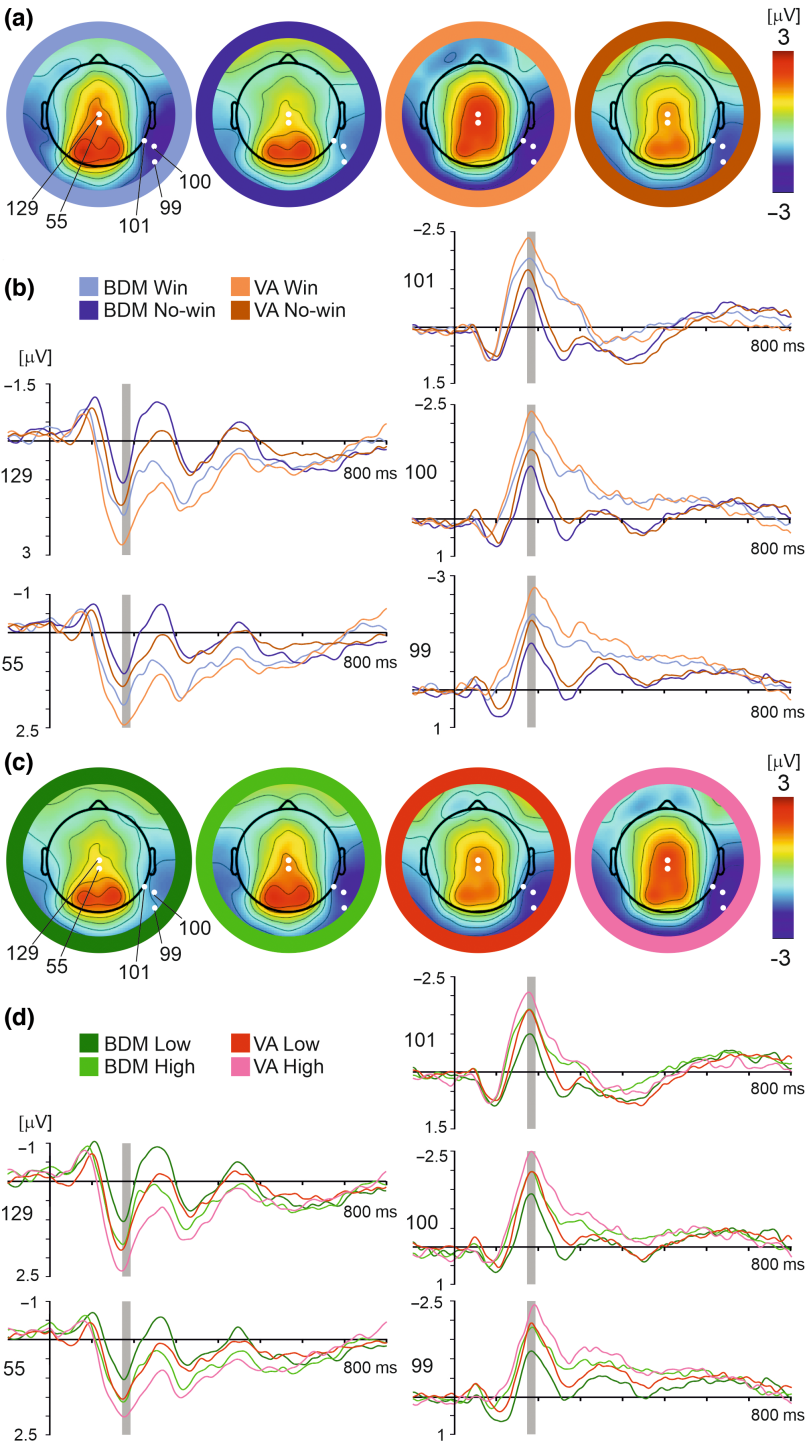
Win–no‐win contrasts (a, b) and high vs. low bid values contrasts (c, d) in BDM and VA tasks for the N170 and the VPP. (a) Whole scalp topographic maps displaying grand average ERPs for each outcome condition at time point 182 ms. Electrodes used in statistical analysis (129, 55, 99, 100, 101) are highlighted in white. (b) Grand average ERP waveforms across all participants and subjective value conditions comparing the four outcome conditions: BDM win (light blue), BDM no‐win (navy), VA win (light orange), and VA no‐win (dark orange). Epoch of interest (172–192 ms post‐feedback‐onset) highlighted in gray. (c) Whole scalp topographic maps displaying grand average ERPs for each value condition at time point 182 ms. (d) Grand average ERP waveform across all participants and outcome conditions comparing the four value conditions: BDM low‐value (dark green), BDM high‐value (light green), VA low‐value (red), and VA high‐value (pink).

For analysis of the N170 component, based on visual inspection of scalp topographic maps (Figure 3) and previous research (Eimer, 2000; Rossion, 2014; Rossion & Jacques, 2012), the epoch of 172–192 ms post‐outcome stimulus onset and the occipitotemporal electrodes 99, 100, and 101 (100 corresponding for TP10 in the 10–10 system) and the vertex electrodes 129 and 55 (corresponding for Cz and CPz in the 10–10 system) were selected for statistical analysis. The ERP waveforms for win and no‐win outcomes and bid value contrasts in both tasks are shown in Figure 3.

As far as the negative potential in occipitotemporal electrodes is concerned, a 3 × 2 × 2 × 2 repeated measures ANOVA (electrode × task × value × outcome) revealed main effects of electrode (*F*(2, 46) = 4.46, *p* = .017, ƞp^2^ = 0.16), outcome (*F*(1, 23) = 12.48, *p* = .002, ƞp^2^ = 0.35), task (*F*(1, 23) = 10.34, *p* = .004, ƞp^2^ = 0.31), and value (*F*(1, 23) = 11.34, *p* = .003, ƞp^2^ = 0.33). No significant interaction effects were found. Post hoc pairwise comparisons showed that: electrode 101 (−1.59 ± 0.23 μV) showed less negative amplitudes than electrode 100 (−2.01 ± 0.27 μV, *p* = .004); win trials (−2.09 ± 0.26 μV) resulted in more negative potential amplitudes than no‐win trials (−1.53 ± 0.23 μV); VA trials (−2.11 ± 0.27 μV) resulted in more negative potential amplitudes than BDM trials (−1.52 ± 0.23 μV); high‐value trials (−1.93 ± 0.24 μV) resulted in more negative potential amplitudes than low‐value trials (−1.70 ± 0.23 μV).

Analysis of the VPP using a repeated measures 2 × 2 × 2 × 2 ANOVA (electrode × task × value × outcome) revealed main effects of task (*F*(1, 23) = 11.53, *p* = .002, ƞp^2^ = 0.33), value (*F*(1, 23) = 4.39, *p* = .047, ƞp^2^ = 0.16), and outcome (*F*(1, 23) = 12.94, *p* = .002, ƞp^2^ = 0.36). There was also a significant interaction effect between task and electrode (*F*(1, 23) = 4.71, *p* = .041, ƞp^2^ = 0.17), and between task and outcome (*F*(1, 23) = 9.23, *p* = .006, ƞp^2^ = 0.29).

Notably, post hoc pairwise comparisons showed that amplitudes for win outcomes were significantly greater in the VA task than in the BDM (VA win: 2.39 ± 0.40 μV, BDM win: 1.50 ± 0.34 μV, *p* < .001), but there was no significant difference between tasks for no‐win outcomes (VA no‐win: 1.37 ± 0.26 μV, BDM no‐win: 1.03 ± 0.24 μV, *p* = .097). Amplitudes between the two electrodes also significantly differed during the VA (129: 1.97 ± 0.34 μV; 55: 1.25 ± 0.31 μV, *p* = .046) but not during the BDM (129: 1.79 ± 0.30 μV; 55: 1.28 ± 0.25 μV, *p* = .805). Further, VA trials (1.88 ± 0.32 μV) resulted in more positive potential amplitudes than BDM trials (1.26 ± 0.28 μV); high‐value trials (1.71 ± 0.29 μV) resulted in more positive potential amplitudes than low‐value trials (1.43 ± 0.29 μV); and win trials (1.94 ± 0.36 μV) resulted in more negative potential amplitudes than no‐win trials (1.20 ± 0.23 μV).

#### 3.2.2 RewP component

**FIGURE 4:**
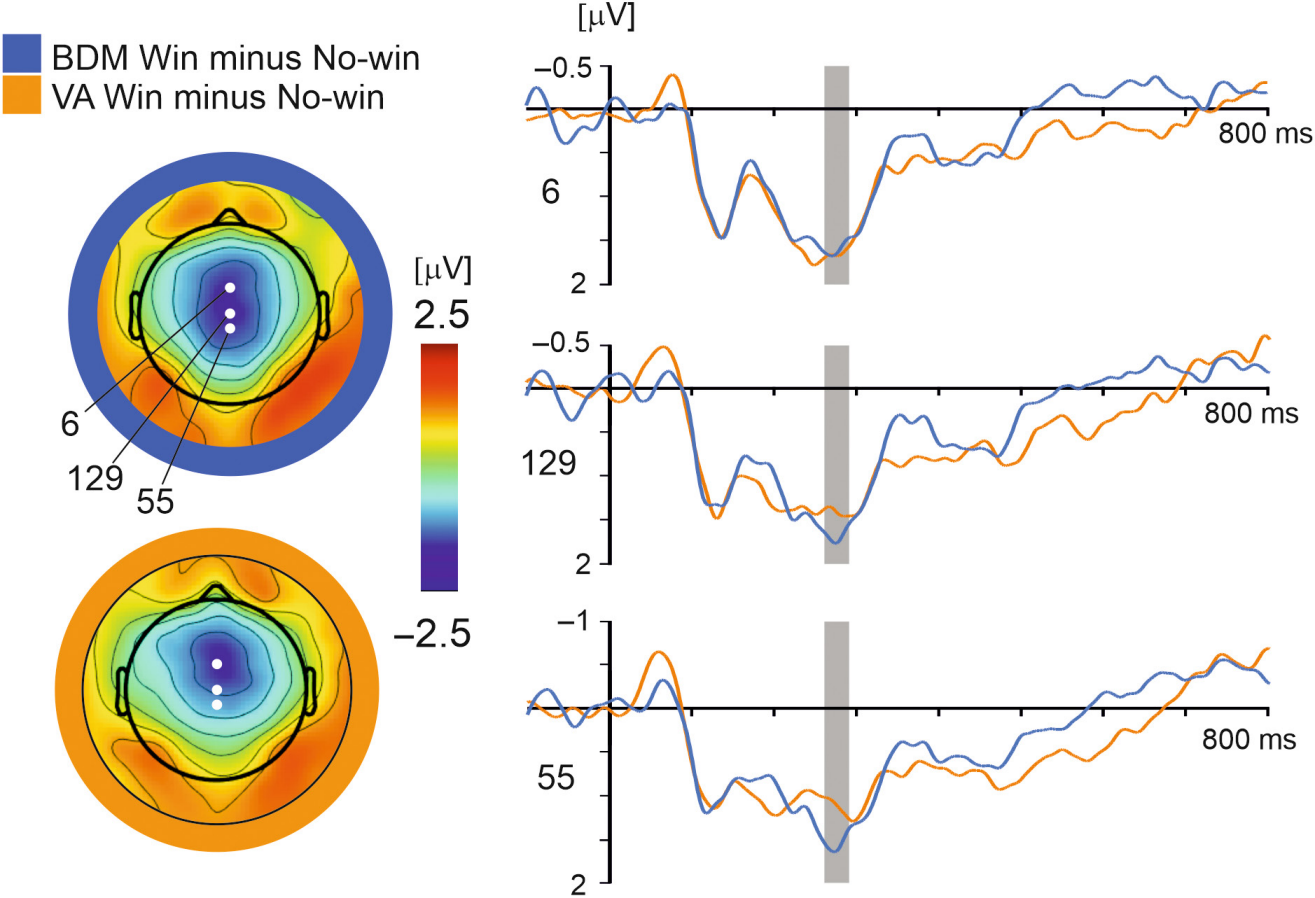
The Win–No‐win contrast in BDM and VA tasks in the RewP component. Left: Whole scalp topographic maps displaying differences in grand average ERPs at time point 275 ms. Three electrodes used in statistical analysis numbered 6, 129, and 55, are highlighted in white. Right: Grand average win–minus–no‐win ERP difference waveform across all subjects and product value conditions comparing BDM (blue) and VA (orange) win–minus–no‐win difference waveforms. Epoch of interest, 260–290 ms post‐feedback‐onset, highlighted in gray.

A RewP with a spatial maximum at the central midline electrodes was found in response to bidding outcomes in VA and BDM during the epoch 260–290 ms. The electrodes 6, 129 and 55 (corresponding to FCz, Cz, and CPz in the 10–10 system) were selected for statistical analysis. The ERP win–minus–no‐win difference waveforms are shown in Figure 4. A 3 × 2 × 2 repeated measures ANOVA (electrode × task × value) revealed no main effects or interaction effects.

#### 3.2.3 P3 component

**FIGURE 5:**
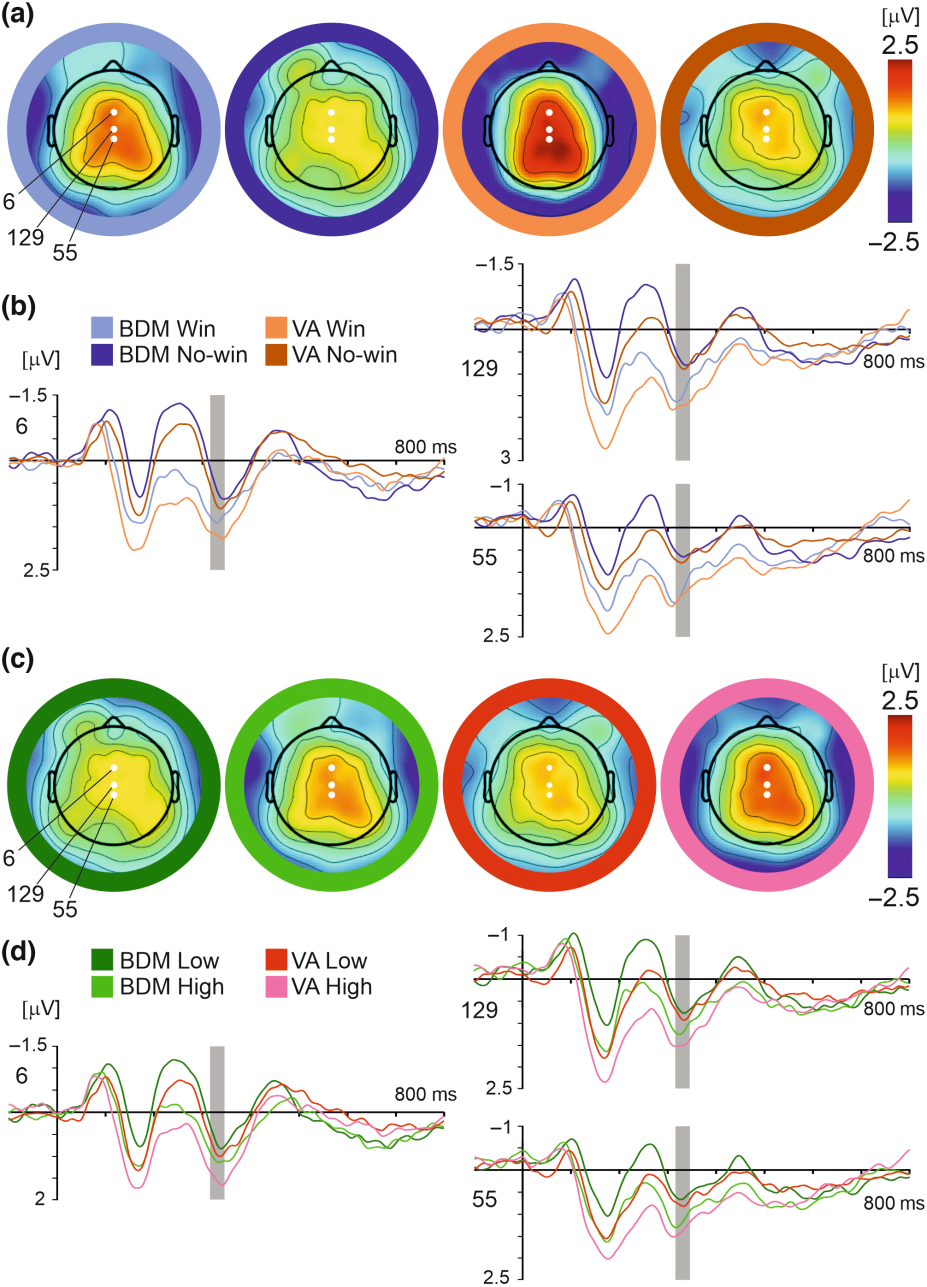
The win–no–win contrast (a, b) and the high vs low‐value bids contrast (c, d) in BDM and VA tasks in the P3 component. (a) Whole scalp topographic maps displaying grand average ERPs for each outcome condition at time point 331 ms. Three electrodes used in statistical analysis numbered 6, 129, and 55, are highlighted in white. (b) Grand average ERP waveforms across all subjects and product value conditions comparing the four outcome conditions: BDM win (light blue), BDM no‐win (navy), VA win (light orange), and VA no‐win (dark orange). Epoch of interest (316–346 ms post‐feedback‐onset) highlighted in gray. (c) Whole scalp topographic maps displaying grand average ERPs for each value condition at time point 331 ms. (d) Grand average ERP waveform across all subjects and outcome conditions comparing the four value conditions: BDM low‐value (dark green), BDM high‐value (light green), VA low‐value (red), and VA high‐value (pink).

Topographic maps of the P3 component for the win–no‐win contrast and the high vs low‐value contrast (Figure 5) showed a positive potential over the midline parietal electrodes, peaking at 316–346 ms. The electrodes 6, 129, and 55 were selected for statistical analysis.

A 3 × 2 × 2 × 2 repeated measures ANOVA (electrode × task × value × outcome) revealed a main effect of value (*F*(1, 23) = 6.83, *p* = .016, ƞp^2^ = 0.23) and outcome (*F*(1, 23) = 6.52, *p* = .018, ƞp^2^ = 0.22), and, importantly, an interaction effect between task and outcome (*F*(1, 23) = 6.35, *p* = .019, ƞp^2^ = 0.22). No other interaction or main effects were found.

Post hoc pairwise comparisons showed that high‐value trials (1.33 ± 0.20 μV) resulted in more positive potential amplitudes than low‐value trials (0.99 ± 0.20 μV), and win trials (1.43 ± 0.24 μV) resulted in more positive potential amplitudes than no‐win trials (0.88 ± 0.19 μV). As far as the interaction between task and outcome is concerned, amplitudes for win outcomes were significantly greater than no‐win outcomes in the VA task (Win: 1.68 ± 0.26 μV; no‐win: 0.85 ± 0.22 μV, *p* = .001) but not in the BDM (Win: 1.18 ± 0.28 μV; no‐win: 0.92 ± 0.25 μV, *p* = .345) (Figure 5).

## 4 DISCUSSION

The present study examined the impact of social competition on economic valuation and the cortical representations of outcome processing during risky economic decision‐making. Two well‐established second‐price sealed‐bid auction paradigms were utilized to elicit SVs and isolate the impact of a second human competitor on reward‐related ERPs. Contrasting the two tasks highlights the impact of a social dimension while comparing win and no‐win outcomes examines the effect of feedback valence, and splitting the data into high and low SV investigates feedback salience. Our results found that the amplitude of the P3 component for win compared to no‐win trials was larger in the VA than in the BDM. Further, the VA task, relative to the BDM, was associated with an unanticipated prominent negative potential in the right occipitotemporal electrodes and a VPP in the latency range of approximately 170–190 ms, suggesting the modulation of a face‐sensitive N170 potential. Both the P3 and N170 were also sensitive to SV, as indexed by bid value, and trial outcome. A RewP, defined as a win–minus–no–win difference waveform, was elicited in both tasks but was not modulated by task or value.

The increased amplitudes of the N170 potential and the VPP in the VA relative to the BDM were not hypothesized. The N170 in the right occipitotemporal electrodes is well established as a face‐sensitive component, traditionally posited to reflect early bottom‐up visual perception (Deffke et al., 2007; Itier & Taylor, 2004). The VPP is a frequent companion to the negative N170 potential (Jeffreys, 1996; Joyce & Rossion, 2005; Rossion, 2014), and the two components are thought to be opposing manifestations of the same brain processes (Zhao et al., 2019). There is evidence that the mental imagery of faces recruits the same early processing mechanisms as face perception (Ganis & Schendan, 2008). The N170 has been shown to be elicited by imagining a face (Dijkstra et al., 2018), and modulated by prompted mental imagery (Ganis & Schendan, 2008) and auditory semantic information (Landau et al., 2010) prior to face perception. These enhancement effects reflect the influence of top‐down processing pathways on the N170 component, with the mental visualization of a person recruiting additional perceptual processing resources. In the present study, the visual stimuli in the outcome period were words and were identical between the two tasks. Nevertheless, the outcome feedback in the VA was not merely financial information, as in the BDM, but also a valid social signal of their opponents' SV. It is possible that the additional mental visualization of a human opponent during the VA task caused a projection of personhood onto the expected incoming visual stimuli, which enhanced the VPP and N170 component amplitudes through top‐down processes. Together, the activations of a prominent VPP and negativity in the occipitotemporal electrodes in the VA present strong evidence that the VA, but not the BDM, activated the fusiform cortices responsible for the N170 face‐sensitive component. This is a preliminary finding, and the VPP and the N170 component were not the focus of this study but merit further exploration in future research.

Further, the P3 component activity also differentiated between the two tasks: P3 amplitudes showed an effect of feedback valence in the VA but not in the BDM. Consistent with prior studies, a P3 was elicited in both tasks over central midline sites peaking at 331 ms post‐outcome feedback presentation, showing a more positive amplitude for win outcomes than no‐win outcomes in the VA. The P3 is well established as sensitive to outcome probability (Duncan‐Johnson, & Donchin, 1982; Polich, 2007, 2012; Polich & Margala, 1997; Rosenfeld et al., 2005) and so a main effect of outcome was expected, as in both tasks no‐win outcomes were more probable than win outcomes. The task parameters of both auctions prescribe that the participants are in control of the likelihood of win/no‐win outcomes. As the bid value directly dictates the outcome probabilities (for example, in this experiment a bid of £3 designates a 25% probability of winning), we were unable to control the respective quantities of win/no‐win trials. However, the overall win/no‐win probabilities in both tasks were comparable, with the average likelihood of winning being 38.83% and 38% for VA and BDM, respectively. The P3 component is central to the allocation of attentional resources based on motivational significance (Nieuwenhuis, Slagter, et al., 2005), therefore the observed increase in amplitude during the VA wins compared to the BDM wins can also be interpreted as reflecting the enhanced stimulus salience of social compared to non‐social feedback stimuli (Bellebaum & Daum, 2008; Gehring & Willoughby, 2002; Pfabigan et al., 2019). The final price paid in VA win trials is the only source of information about the opponent and their SVs, and so can be utilized as a valid social signal for the participant to learn about the public, shared values of items.

No other ERP component in the present study differentiated between VA and BDM tasks. Specifically, the lack of RewP component sensitivity to the social domain in this competitive context is interesting when compared to previous research. In the present study, the level of competition remained the same, but the source of competition was changed. In previous studies where RewP amplitudes were modulated in social competitive scenarios (Czeszumski et al., 2019; Luo et al., 2015), the level or type of competition (e.g., direct or indirect) was altered while the social context remained the same. Our results align with those found by Rigoni et al. (2010), where the RewP amplitudes did not differ between a non‐social and a socially competitive context. Together, these results suggest that the type of competition, for example, comparison vs direct competition, is the modulating factor in RewP amplitude. Further studies could more comprehensively unpack the relationship between competition/comparison type and social context to elucidate the respective contributions to RewP activity.

The amplitudes of the N170 and P3 components, but not the RewP, were modulated by SV in both types of auctions, with greater amplitudes for high‐ than low‐value auction items. The insensitivity of the RewP to SV as dictated by bid value is in line with findings from our previous VA study, where the RewP was indifferent to the market value of the auction items (Newton‐Fenner et al., 2022). The more positive P3 amplitude potentials following high SV outcomes compared to low SV items may be linked to attentional engagement (Nieuwenhuis, Aston‐Jones, et al., 2005; San Martin, 2012; Yeung & Sanfey, 2004). Our results indicate that high‐value item trials were deemed more salient and consequently garnered greater attentional resources in the outcome processing period. The participants were more invested in the outcomes of the trials where they had bid higher, and this increase in engagement was reflected in the initial neural response.

Finally, it is important to note that, while this study builds on previous research conducted in our lab and provides new insights into outcome processing in risky economic contexts, it is also limited by a potential confound in the outcome stimuli. The additional information when items were won resulted in a luminance difference between win and no‐win outcomes. While this was necessary to convey the details of the final price paid to the participant, it may have impacted early low‐level visual components. Our previous VA study found an impact of final price paid in win trials on RewP but not P3 amplitude, so future studies could explore the interaction of social context and the final price paid to further understand this information's role as a valid social signal of shared SV.

To conclude, the present study showed that two event‐related components differentiated between Vickrey and BDM auctions. An unanticipated N170 component, and P3 amplitudes for high‐value items, were enhanced in the VA compared to the BDM. Findings suggest that automatic feedback processing as early as 176 ms post‐feedback onset is facilitated by the presence of a human competitor and that later processing of outcome feedback is modulated by social context and subjective value. Further, our results align with previous investigations that found reward‐related components are differentially sensitive to outcome valence and salience. This study progresses the neural characterization of the impact of social context on reward processing in risky environments.

## AUTHOR CONTRIBUTIONS

**A. Newton‐Fenner:** Conceptualization; data curation; formal analysis; investigation; methodology; project administration; writing – original draft; writing – review and editing. **D. Hewitt:** Investigation; writing – review and editing. **J. Henderson:** Investigation; software; writing – review and editing. **N. Fallon:** Conceptualization; supervision; writing – review and editing. **Y. Gu:** Conceptualization; supervision; writing – review and editing. **O. Gorelkina:** Conceptualization; supervision; writing – review and editing. **T. Giesbrecht:** Conceptualization; funding acquisition; supervision; writing – review and editing. **A. Stancak:** Conceptualization; funding acquisition; software; supervision; writing – review and editing.

## FUNDING INFORMATION

This work was supported by the EPSRC and ESRC Centre for Doctoral Training [Grant Number: EP/L015927/1] on the Quantification and Management of Risk and Uncertainty in Complex Systems and Environments, University of Liverpool, Liverpool, UK, and Unilever.

## CONFLICT OF INTEREST STATEMENT

The authors declare that they have no conflict of interest.

## Supporting information


Figure S1

## Data Availability

Data and MATLAB files are available on the open‐access platform OSF: https://osf.io/2d7ub/?view_only=995efddf256d4af5ad6f945249eef3e3
