# Supplementary material for: A comparison of reward processing during Becker–DeGroot–Marschak and Vickrey auctions: An ERP study
Source: Psychophysiology. 2023 Apr 19;60(9):e14313. doi: 10.1111/psyp.14313 (PMC10909440; doi:10.1111/psyp.14313)

***Supplementary Materials***

The topographic maps of the VPP and N170 components overlaid on the 3D volume rendering of a human head, shown at time point 176ms post-feedback onset.


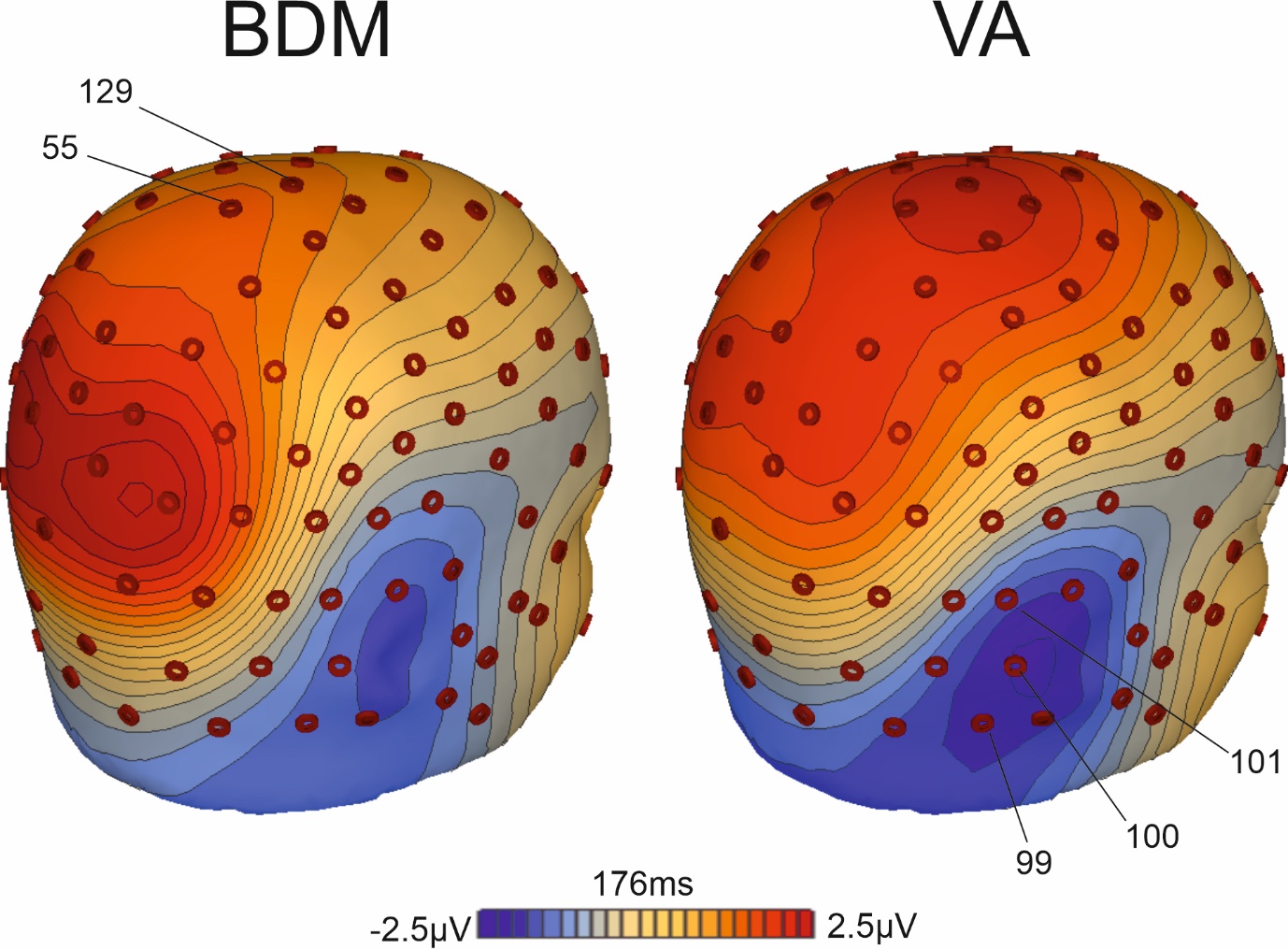

Supplement: Supplementary file 1 — Figure S1 [file PSYP-60-e14313-s001.docx]
